# Artificial intelligence applications in cardio-oncology: Leveraging high dimensional cardiovascular data

**DOI:** 10.3389/fcvm.2022.941148

**Published:** 2022-07-26

**Authors:** Haidee Chen, David Ouyang, Tina Baykaner, Faizi Jamal, Paul Cheng, June-Wha Rhee

**Affiliations:** ^1^City of Hope National Medical Center, Duarte, CA, United States; ^2^Cedars Sinai Medical Center, Los Angeles, CA, United States; ^3^Department of Medicine, Division of Cardiovascular Medicine, Stanford University, Palo Alto, CA, United States

**Keywords:** cardio-oncology, artificial intelligence, echocardiography, electrocardiogram, imaging

## Abstract

Growing evidence suggests a wide spectrum of potential cardiovascular complications following cancer therapies, leading to an urgent need for better risk-stratifying and disease screening in patients undergoing oncological treatment. As many cancer patients undergo frequent surveillance through imaging as well as other diagnostic testing, there is a wealth of information that can be utilized to assess one's risk for cardiovascular complications of cancer therapies. Over the past decade, there have been remarkable advances in applying artificial intelligence (AI) to analyze cardiovascular data obtained from electrocardiograms, echocardiograms, computed tomography, and cardiac magnetic resonance imaging to detect early signs or future risk of cardiovascular diseases. Studies have shown AI-guided cardiovascular image analysis can accurately, reliably and inexpensively identify and quantify cardiovascular risk, leading to better detection of at-risk or disease features, which may open preventive and therapeutic opportunities in cardio-oncology. In this perspective, we discuss the potential for the use of AI in analyzing cardiovascular data to identify cancer patients at risk for cardiovascular complications early in treatment which would allow for rapid intervention to prevent adverse cardiovascular outcomes.

## Introduction

Cardio-oncology is a new frontier in cardiology that aims to improve the prevention, detection, and management of cardiovascular complications caused by cancer therapies of various mechanisms (1). As complications of cancer therapies can both compromise oncologic and cardiovascular outcomes, it is imperative to identify cohorts susceptible to developing cardiotoxicity to oncologic treatments and detect cardiotoxicity at the earliest possible time point to allow for alteration of treatments or implementation of cardioprotective strategies (2).

**Figure 1:**
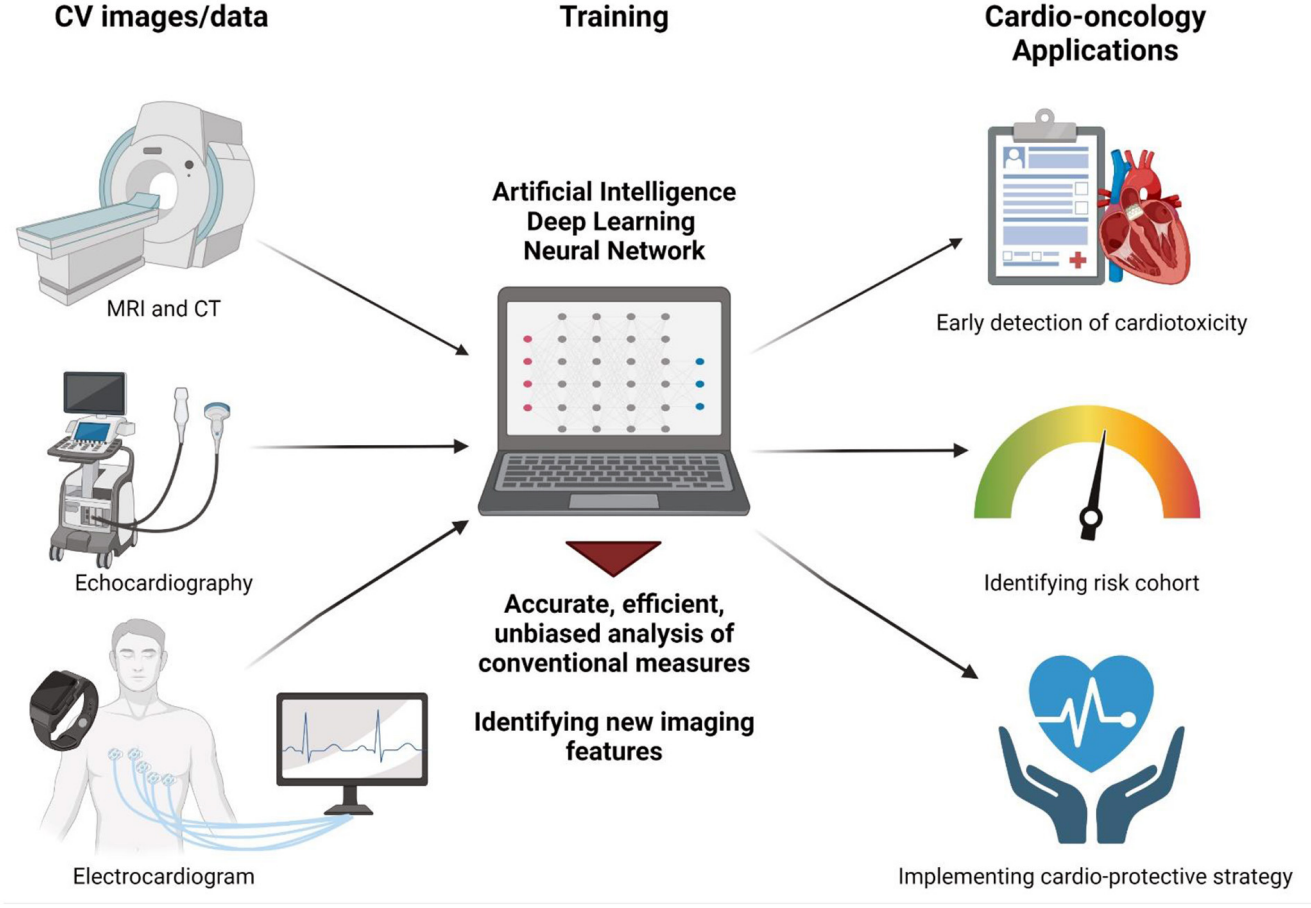
Artificial intelligence applications in cardio-oncology. AI-enabled analysis of routinely collected cardiovascular images such as MRI, CT, echocardiography, and electrocardiogram may facilitate (1) accurate, efficient, unbiased analysis of conventional measures such as LVEF and (2) identification of new image features not previously recognized to correlate with cardiotoxicity. This will ultimately help physicians to detect early signs of cardiotoxicity, identify at-risk cohorts, and implement cardioprotective strategies early on to optimize cardiovascular health of cancer patients and thereby allow safe and effective cancer treatments. Figure created with Biorender.

Recent advances in applying artificial intelligence (AI) to routinely obtained cardiovascular data have demonstrated the ability of these sophisticated algorithms to both risk-stratify patients from simple sets of data as well as drastically improve the detection for a variety of different conditions (3). These promising studies raise the tantalizing potential of AI to revolutionize the field of cardio-oncology and facilitate the identification of and protection against unwanted cancer therapy-associated cardiotoxicity. In this perspective, we review currently available recent breakthroughs in using AI in cardiovascular imaging and how this may be applied in our cardio-oncology population to better monitor, assess, and diagnose their underlying cardiovascular conditions, thereby minimizing cardiovascular toxicity while maximizing cancer therapeutic efficacy (Figure 1).

## AI application in early detection of myocardial toxicity

Cardiac dysfunction has been associated with a wide spectrum of cancer therapies including but not limited to anthracyclines, HER2 targeted therapies, select tyrosine kinase inhibitors (TKIs), radiation therapy, and more recently immunotherapies especially in association with myocarditis (2). Clinical manifestations of cancer therapy-related cardiac dysfunction can vary significantly ranging from asymptomatic left ventricular (LV) dysfunction to heart failure, and potentially fatal cardiogenic shock. Albeit with conflicting data, numerous studies have suggested benefits of early initiation of neurohormonal blockade to prevent LV function deterioration (4). However, the implementation of such strategies has been limited in part by rather modest beneficiary effects seen in the studies. Early detection of cardiac dysfunction may provide an opportunity to initiate cardioprotective intervention in a timely manner to derive a maximal benefit and optimize cardiovascular health and screening strategy to minimize subsequent toxicity. AI-guided analysis of routinely collected cardiovascular images may help identify patients with early cardiotoxicity.

### Early detection of cardiac dysfunction

**Left ventricular ejection fraction (LVEF)** changes have been primarily used to diagnose cardiotoxicity of cancer therapies. The American Society of Echocardiography and the European Association of Cardiovascular Imaging guidelines recommend averaging five consecutive beats to calculate LVEF (5, 6). However in practice, as the measurement and calculation can be time consuming, generally one representative beat is used to estimate LVEF. This can lead to inaccuracies and variation in quantifying LVEF as it can have significant variability from beat to beat. Additionally, small changes in a variety of technical factors frequently cause large changes in the measurement of LVEF (7). Technical differences in image acquisition, variability between operators performing cardiac ultrasound, and subjective differences in appreciation of the blood-tissue interface between readers collectively contribute to variability in measurements of LVEF (8). Inter-reader variability in reporting of LVEF has been reported as high as 10% (9). Incidentally, a reduction of 10% in ejection fraction is the same magnitude of change that is used to define significant cardiotoxicity, resulting in potential need to interrupt chemotherapy. This level of inter-reader variability presents a significant challenge in cardio-oncology. Furthermore, small drops in LVEF are often attributed to measurement noise rather than a true signal of toxicity, often leading to delayed detection of cardiotoxicity. The answer to these challenges lies in automation. While imaging software has improved in the past several decades to include automated contouring of the left ventricle, present software packages generally fail to evaluate the endocardial border with sufficient accuracy in technically difficult studies with suboptimal images. The most promising solution to minimize inter-reader variability and improve reproducibility of cardiac function assessments is the evolution of AI. In a recent study, Ouyang and colleagues show that through deep-learning, LVEF can be more accurately and unbiasedly measured with variance that matches or is less than that of human specialists (8). The use of AI allows for quicker segmentation and beat to beat quantification, facilitating a more efficient, consistent, and accurate quantification of LVEF. The ability to obtain more precise LVEF measurements can allow for meaningful detection of drop in LVEF earlier in the clinical course in an unbiased fashion. These will then allow for more efficient identification of those who will likely benefit from early initiation of cardioprotective therapies.

**Global longitudinal strain (GLS)** has been proposed as a more sensitive means for early detection of cardiac dysfunction prior to a detectable drop in LVEF. A consistent measurement of left ventricular GLS could be instrumental in guiding chemotherapeutic regimen. However, the study by Farsalinos and colleagues noted significant variability among various ultrasound system vendors when measuring GLS (10). While GLS may be more reproducible and relatively easier to obtain compared to LVEF, these inter-vendor differences can influence results and the applicability of GLS among different practices. To address this, Kwan and colleagues demonstrated that an automated deep-learning strain (DLS) pipeline could help to reconcile these differences and result in better inter-vendor agreement regardless of subjective image quality (11). Thus, the use of AI can help facilitate the measurement of strain, resulting in a more efficient, standardized, and vendor-blind method across images of varying qualities. Additionally in a separate study, Salte and colleagues demonstrated a similar result in reducing measurement variation and providing a more efficient way to quantify strain (12). AI-enabled, more universal and consistent GLS measurement across vendors may help identify subtle, early signs of cardiotoxicity by cancer therapies that may be too early to detectably alter LVEF.

### AI to decode cardiovascular image features to correlate cardiotoxicity

Hughes and colleagues utilize AI in analyzing echocardiographic images to detect abnormalities in a variety of cardiac blood biomarkers such as B-type natriuretic peptide (BNP), troponin I, and blood urea nitrogen (BUN) (13, 14). Through analysis of the echocardiography videos, they were able to show that their EchoNet-Labs model estimated biomarkers of cardiac function with a high degree of specificity and sensitivity across diverse racial and ethnic groups. Additionally, they show that across two separate institutions, the model demonstrated similar sensitivity and specificity for both patient populations. This result further strengthens the idea that AI can be used to standardize and accurately risk stratify patient populations correlating blood markers for cardiovascular diseases and complications across institutions and between physicians.

Similarly, AI can identify less obvious information contained in EKG images to detect conditions like anemia which may further assist in identifying risk cohorts (15). EKGs are an imaging tool that is both inexpensive and ubiquitous in healthcare settings. Beyond the conventional parameters being measured such as PR/QT intervals, AI-guided EKG image analysis may provide unrecognized patterns to predict clinical phenotypes. As an example, anemia is usually difficult to diagnose with patient histories and physical exams only, necessitating blood draws. Kwon and colleagues demonstrate that AI analysis of EKGs can provide an alternative less-invasive and less-costly method of detecting anemia (15). Beyond anemia, as we accumulate more data to correlate with other disease biomarkers of cardiac complications (e.g., inflammatory complication such as myocarditis) (16), there may be unprecedented opportunities to utilize these studies to predict disease course and guide our management.

Furthermore, AI in EKG may help identify early signs of myocardial dysfunction caused by cancer therapies. Attia et al. reported that AI-guided analysis of EKG accurately and reliably identified asymptomatic LV dysfunction defined as ejection fraction ≤ 35% with sensitivity and specificity of 86.3 and 85.7% respectively (17). In a separate study, Adedinsewo and colleagues demonstrated the efficacy of using AI to identify dyspneic patients at risk of systolic heart failure in the emergency department (18). They showed that AI analysis of EKGs allowed accurate identification of LV systolic dysfunction and even outperformed N-terminal pro-B-type natriuretic peptide (NT-proBNP) measurements. Additionally, they showed that their AI-enabled EKG algorithm is both inexpensive and efficient in recognizing dyspneic ED patients with LVSD compared to existing diagnostic methods like echocardiography, which is operator-dependent and requires trained specialists, and the measurement of NT-proBNP alone (18). Identification of cancer patients similarly at risk of LV dysfunction using this algorithm that simply leverages already obtained EKG images can expedite subsequent testing and additional medical interventions. As EKGs can be recorded and obtained in the office in a convenient manner, compared to other imaging modalities, it may also be used as a convenient screening tool for those being treated with cancer therapy to identify early signs of myocardial dysfunction. These patients could then be initiated on cardioprotective therapies to minimize their risk of LV function deterioration (19).

## AI applications in predicting drug-induced arrhythmia

Just as EKGs are useful in identifying those at risk for LV dysfunction, paired with AI analysis, they can have the potential to provide low cost and non-invasive screening for atrial fibrillation (AF) as shown by Attia and colleagues (20). AF is often under-detected, associated with an increased risk of cardiovascular events like stroke and heart failure, and can significantly complicate the management of cardio-oncology patients (21, 22). Attia and colleagues have shown that AI can accurately identify patients with a history of AF from their EKGs obtained in normal sinus rhythm (20). Similarly, Tang et al. also demonstrated that EKGs obtained in sinus rhythm analyzed with AI can accurately predict patients who would have favorable outcomes following AF ablation (23). These studies suggest potential signatures of rhythm disorders such as AF in EKGs obtained in normal sinus rhythm, that are not apparent and easily analyzable by traditional signal processing and statistical methods. They also bring along the possibility of screening cancer patients with EKGs to determine who may be at higher risk of AF, and may benefit from frequent screening to help manage and minimize AF related complications.

In addition to identifying those who might be at risk for AF, deep learning analysis of EKGs can be used to assess patients at risk for drug-induced QT prolongation (24). QT prolongations may lead to fatal arrhythmias like torsade de pointes (TdP) that can lead to sudden cardiac death. Through identifying a patient at risk for drug-induced QT prolongation and subsequent TdP, immediate intervention can be implemented to prevent a fatal outcome. Prifti and colleagues have shown that use of convolutional neural network models to analyze EKGs more accurately screen for risk of drug-induced TdP than the current standard of measuring QT interval corrected for heart rate (25). In the field of cardio-oncology, screening for those at risk of developing QT prolongation and fatal arrhythmias before they develop is critical as many cancer treatments such as tyrosine kinase inhibitors are associated with QT prolongation (26).

## AI applications in identifying at-risk cohort for cardiotoxicity

Preexisting cardiovascular diseases (CVD) and risk factors have been associated with an increased likelihood of subsequent cardiotoxicity following cancer therapies (27). Therefore, precise assessment of one's pre-existing CVD and relevant risk factors is critical in assessing one's future CV risk following cancer therapies. Cardiovascular disease screening prior to cancer therapies can be done through using many metrics such as laboratory biomarkers, coronary artery calcium (CAC) scoring from CT imaging for CAD assessment, LVEF by echocardiogram, and EKG. Additionally, recent research suggests that deep-learning analysis of other routinely collected image studies during cancer diagnosis can be leveraged for further evaluation.

Coronary artery disease (CAD) and cancer are both diseases found more frequently in the elderly. Presence of significant CAD increases the risk of treatment-associated complications, and the evaluations and treatment of these CAD, such as stress testing and angiograms frequently create delays for definitive therapy. A rapid evaluation of a patient's CAD risk at the time of cancer diagnosis has the potential to streamline the process and deliver timely care. One commonality among oncological patients is routinely performed cross-sectional imaging such as CT for cancer staging. Staging CT scans are traditionally not felt to be sufficient to evaluate one's risk of CAD as they are not cardiac-gated. The application of AI to non-gated imaging has recently been shown to be able to generate accurate CAC score predictions (28, 29). This approach will allow for CAD diagnosis and risk stratification to be performed at no additional cost or radiation. It is likely, in fact, further algorithms can be developed to utilize serial staging CTs to closely monitor and predict patient's risk for cardiovascular complications. Furthermore, AI has been shown to rapidly and accurately assess CAD progression and prognosis through analysis of plaque volume and stenosis severity acquired from coronary CT angiography (CCTA) images, and these assessments closely align with those of expert readers (28). These findings could have an important implication in identifying risk for future cardiovascular diseases like myocardial infarctions, which would help to direct future treatments and screening.

In another study, Poplin and colleagues used deep-learning models to analyze retinal fundus images and predict cardiovascular risk factors that were not previously thought to be quantifiable or manifested in retinal images, like age, gender, blood pressure, and ethnicity (13). Currently, information from a patient's history or blood tests are used as the standard for assessing cardiovascular risk (30). Poplin and colleagues' model achieved similar precision in identifying cardiovascular risk as the Systemic Coronary Risk Evaluation calculator and their results suggest a promising role for deep learning in assessing cardiovascular risk factors and monitoring how well one's hypertension or diabetes is controlled through less invasive imaging and analysis.

Finally, Attia and colleagues have shown that AI-guided analysis of EKGs could identify patients who had normal LVEF and were at a four-fold risk of developing left ventricular dysfunction over the next 5 years and before apparent left ventricular dysfunction could manifest symptomatically (17). Furthermore, it was found that the AI performed better across all ages and sexes. Thus with the identification of at risk populations, one can take preventative measures and monitor patients more closely to encourage better health outcomes.

## Limitations and specifical consideration

Although the prospect of utilizing AI and deep learning to predict patients' risks of developing cardiotoxicity from cancer treatments is exciting and promising, there are practical hurdles and methodological limitations that currently prevent these techniques to reach their full potential. Prospective validation of the utility of incorporating the AI-mediated clinical decision-making tool into routine clinical practice is needed prior to the implementation of these promising avenues into cardio-oncology practice. For this, our institutions are collaboratively working toward building a prospective, well-labeled database with predefined variables to employ available AI-based tools in analyzing cardiovascular data to predict future cardiovascular outcomes. However, there remain challenges due to the heterogeneity of treatment regimen, diverse patient populations, differences in mechanism of toxicities, and relatively small cohorts. Such challenges may preclude effective development and validations of these AI tools, and must be overcome in order for these powerful tools to reach their full potential. International, multi-center collaborations to build well-curated patient data registry, such as the recent initiative by International CardioOncology Society, may help overcome these hurdles to identify meaningful signals among these heterogeneous populations (31). Finally, intentional efforts to educate and engage broader cardio-oncology communities early on and disseminate these tools, once validated, would be critical to derive maximal benefits of these advances in managing cardiovascular health of cancer patients.

## Conclusion and future directions

The use of AI in detecting vulnerable populations for cardiovascular complications due to cancer treatment is extremely important and timely in the field of cardio-oncology and has the potential to greatly improve patient care. Leveraging recent advances in AI to analyze patient images and data standardizes the procedure, generating more consistent results that don't vary from physician to physician and reduce human error. The use of AI would not only improve the accuracy of measurements such as LVEF, but it would also identify noble imaging features correlated with cardiotoxicity. With better identification of at-risk populations or patients with early signs of cardiotoxicity, clinical care can be optimized for each patient to implement cardioprotective strategies early on in treatment to achieve better cardiac and oncologic outcomes.

## Data availability statement

The original contributions presented in the study are included in the article/supplementary material, further inquiries can be directed to the corresponding authors.

## Author contributions

HC, DO, PC, and J-WR conceived and outlined the manuscript. FJ and TB contributed to design the study and provided critical input on the manuscript. HC, PC, and J-WR wrote the manuscript with input from all authors. All authors contributed to the article and approved the submitted version.

## Funding

This work is supported by of National Institutes Health grants K08 HL148540 (J-WR), K08 HL153798 (PC), K23 HL145017 (TB), K99 5K99HL157421, and American Heart Association Career Development Awards (J-WR and PC).

## Conflict of interest

Author TB has received speaker and consultant fees from Biotronik, Medtronic, and PaceMate unrelated to this work. Author DO has received consultant fees from Ultromics, Echo.IQ, Anthem, and Alexion. Author FJ has received consultant fees from Janssen unrelated to this work. The remaining authors declare that the research was conducted in the absence of any commercial or financial relationships that could be construed as a potential conflict of interest.

## Publisher's note

All claims expressed in this article are solely those of the authors and do not necessarily represent those of their affiliated organizations, or those of the publisher, the editors and the reviewers. Any product that may be evaluated in this article, or claim that may be made by its manufacturer, is not guaranteed or endorsed by the publisher.
